# Defect-mediated generation–recombination dynamics governing conductance response in floating-body transistors

**DOI:** 10.1038/s41378-026-01393-z

**Published:** 2026-08-04

**Authors:** Been Kwak, Changhyeon Han, Hwoibin You, Wongi Hong, Joonhyeok Choi, Youngchan Cho, Sangwan Kim, Wonjun Shin, Daewoong Kwon

**Affiliations:** 1 Department of Electrical Engineering, Hanyang University, Seoul, 04763 Republic of Korea; 2 Department of Semiconductor Convergence Engineering, Sungkyunkwan University, Suwon, 16419 Republic of Korea; 3 Department of Electrical and Computer Engineering, Sungkyunkwan University, Suwon, 16419 Republic of Korea

**Keywords:** Electrical and electronic engineering, Electronic devices

## Abstract

As CMOS technologies evolve toward ultra-thin floating-body architectures, defect-mediated carrier dynamics increasingly dominate device behavior, challenging conventional linear interpretations of electrical response in nanoscale transistors. In this work, we demonstrate that the conductance response of foundry-fabricated floating-body transistors is governed by generation–recombination (GR) dynamics associated with deep-level defects inside the ultra-thin silicon body. Frequency-dependent conductance measurements reveal bell-shaped responses that exhibit weak gate-bias dependence but strong temperature sensitivity, deviating fundamentally from conventional interface-trap-dominated behavior observed in bulk MOSFETs. By correlating conductance analysis with temperature-dependent low-frequency noise spectroscopy, we identify a common dynamical origin of both phenomena and extract the energetic position and density of channel defects. The conductance peak dynamics follow thermally activated GR processes, indicating that the measured admittance reflects a complex interaction between inversion carriers and channel defects under volume-inversion conditions. Furthermore, electrical stress induces a bias-dependent transition in the dominant conductance mechanism, revealing a crossover from interface-controlled to channel-defect-controlled dynamics. These findings indicate that conductance measurements in ultra-thin floating-body transistors probe a coupled defect system, providing a physically grounded framework for interpreting conductance and noise dynamics in advanced nanoscale electronic devices.

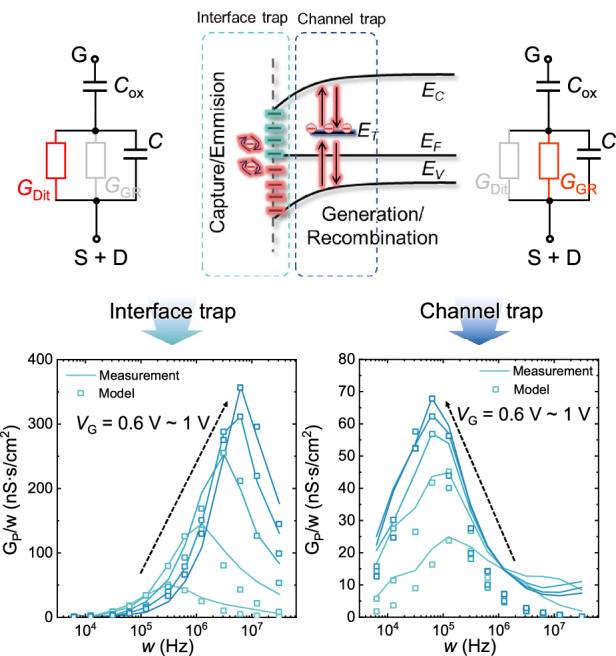

## Introduction

Continued scaling of Complementary metal–oxide–**s**emiconductor (CMOS) logic devices has driven a fundamental transition in transistor architectures toward ultra-thin channel structures, such as gate all around nanosheet transistors^1,2^. In these devices, aggressive reduction of the channel thickness is essential to achieve strong electrostatic control, suppress short channel effects, and maintain acceptable subthreshold characteristics at deeply scaled technology nodes^3^. As a result, the silicon channel is no longer treated as a quasi-bulk region but instead behaves as a nanoscale body whose electrostatics and carrier transport are strongly influenced by confinement effects^4^. A similar scaling trend is observed in advanced dynamic random access memory technologies, where vertical channel transistors are increasingly adopted^5–7^. In these devices, the channel thickness is also aggressively reduced to improve device reliability, suppress leakage currents, and mitigate floating body-related instabilities. Consequently, both advanced logic and memory transistors share a common structural feature: the absence of body contact^8–10^. This intrinsically floating body configuration fundamentally alters carrier dynamics and defect-related phenomena compared to conventional bulk MOSFETs^11^.

For floating-body devices, characterization of interface trap density becomes particularly challenging. Conventional charge pumping techniques rely on periodic modulation of the gate terminal bias to force carrier trapping/detrapping at the oxide semiconductor interface, enabling quantitative extraction of interface trap density using body current^12–14^. However, in the absence of an accessible body terminal, charge pumping measurements cannot be directly applied. This limitation becomes increasingly critical as floating-body architectures are no longer limited to niche technologies but instead form the backbone of modern logic and memory devices.

To overcome this limitation, the conductance method has been widely adopted as an alternative approach for extracting interface trap density in floating-body devices^15,16^. By analyzing the frequency dependent conductance response of a transistor, the conductance method probes carrier capture and emission processes at interface traps through their characteristic energy and time dependent dynamics. Importantly, this method does not require a body terminal, making it particularly attractive for SOI based and multi gate transistor architectures. As a result, the conductance method has often been regarded as a reliable and general technique for interface trap characterization in floating-body devices^17,18^.

The physical basis of the conductance method is well established in conventional bulk MOSFETs. Interface traps exchange carriers with the channel through capture and emission processes, introducing an additional loss component in the small signal admittance. In bulk devices, the peak magnitude of the frequency dependent conductance response is directly proportional to the interface trap density, while the peak frequency is determined by the trap time constant^19^. Under these conditions, the conductance response is predominantly governed by interface related trapping processes, and its interpretation in terms of interface trap density is well understood and widely accepted.

However, when the silicon channel thickness is scaled into the fully depleted regime, additional physical mechanisms become increasingly significant. In ultra-thin channels, microscopic fluctuations of inversion carriers are no longer governed solely by trapping/detrapping processes at the oxide semiconductor interface^20^. Instead, defects located inside the silicon channel itself can actively participate in carrier generation and recombination (GR) processes^21–23^. These channel related defects introduce additional fluctuation pathways that can strongly influence both current noise and small signal conductance responses^24–26^.

In floating-body fully depleted SOI (FD-SOI) transistors, the coexistence of interface traps, channel defects, and floating body effects fundamentally complicate the interpretation of the conductance method. The measured frequency dependent conductance response may no longer originate exclusively from interface trapping processes but instead reflect a complex superposition of interface related and channel related defect dynamics. Despite the increasing technological importance of fully depleted floating-body devices, a systematic investigation of how channel defects affect the validity and interpretation of the conductance method has not yet been reported. This lack of understanding raises critical questions regarding the direct applicability of the conventional conductance method for reliable interface trap density extraction in FD-SOI transistors.

In this work, we systematically investigate the applicability and limitations of the conductance method for interface trap density extraction in FD-SOI transistors, with particular emphasis on the role of channel defects. Investigating 12-inch wafer-scale FD-SOI transistors fabricated in a commercial foundry process, temperature- and bias-dependent low-frequency noise (LFN) spectroscopy is employed to identify GR processes originating from deep-level defects inside the ultra-thin silicon channel and to quantitatively extract their density and energetic position, while carrier number fluctuation (CNF) analysis enables evaluation of the trap volume density (*N*_T_) in the gate dielectric. By directly correlating the conductance response with independently identified noise mechanisms, we demonstrate that channel defects can introduce conductance features that are not accounted for in the conventional interface-trap-only framework. These results reveal that, in floating-body FD-SOI devices, the conductance method may capture a superposition of interface and channel defect dynamics, leading to potential overestimation or misinterpretation of interface trap density if channel effects are neglected. Our study establishes a physically grounded framework for interpreting conductance measurements in ultra-thin floating-body transistors and provides critical guidelines for reliable interface trap characterization in advanced CMOS logic and memory technologies.

## Methods/Experimental

### Device fabrication

The FD-SOI transistor was fabricated on a 300 mm SOI wafer featuring a 9 nm ultra-thin silicon body and 200 nm buried oxide (BOX) thickness. The device active regions were first defined by forming mesa structures through photolithography and dry etching, followed by standard RCA cleaning. A gate stack composed of 5 nm SiO_2_ and a 50 nm phosphorus-doped poly-Si layer was then deposited using atomic layer deposition (ALD) at 330 °C and low-pressure chemical vapor deposition (LPCVD) at 620 °C, respectively. After gate patterning by photolithography and dry etching, self-aligned source/drain regions were formed via phosphorus ion implantation with a dose of 1 × 10^15 ^cm^-2^ at 20 keV under a tilt angle of 7°. Dopant activation was subsequently carried out at 900 °C for 30 s in an N2 ambient using rapid thermal annealing (RTA). A 300 nm SiO_2_ passivation layer was then deposited by plasma-enhanced chemical vapor deposition (PECVD). Finally, after opening the contact windows for the source/drain and gate terminals, Ti/TiN/Al metallization was performed, and the metal patterns were defined using dry etching.

### Electrical measurement system

To evaluate the intrinsic switching characteristics of the device, DC current–voltage (*I*–*V*) measurements were carried out using an Agilent B1500A semiconductor parameter analyzer equipped with precision source/measure unit (SMU) modules. Furthermore, to intentionally induce electrical degradation in the FD-SOI device, periodic gate-pulse cycling was applied using a source pulse generator unit (SPGU, B1525A), enabling controlled stress conditions.

### Temperature control system

For temperature-dependent characterization, the device under test was placed on a temperature-controlled hot chuck. Before each measurement, the chuck was allowed to stabilize at the target temperature, and an adequate settling time was provided to ensure uniform thermal distribution across the device. This procedure minimized temperature gradients and ensured that the entire device reached thermal equilibrium prior to electrical testing. As a result, stable and reproducible measurement conditions were maintained over the investigated temperature range of 20–100 °C.

### LFN measurement

To examine the LFN characteristics of the FD-SOI device, a dedicated measurement setup was employed, consisting of a semiconductor parameter analyzer (B1500A), a low-noise current preamplifier (SR570), and a dynamic signal analyzer (35670 A). This configuration was designed to accurately capture drain-current fluctuations and to obtain the corresponding power spectral density (PSD) through Fast Fourier Transform (FFT) processing. During the measurements, the gate and drain biases were supplied by the B1500A to precisely define the device operating condition, while the SR570 amplified the drain-current signal to enhance the signal-to-noise ratio. All LFN measurements were performed at a fixed drain voltage of *V*_DS_ = 0.05 V, and the gate voltage was adjusted to set the target drain-current levels in the subthreshold-swing (SS) region. The amplified signal was then delivered to the 35670 A, where the time-domain current fluctuations were converted into frequency-domain PSD data using FFT analysis. This method was adopted because, in field-effect transistors, drain-current fluctuations constitute the primary noise component, and their spectral characteristics directly reflect the underlying physical mechanisms, such as GR processes and trap-related carrier-number fluctuations. To maintain accuracy over a broad frequency range, the PSD was measured in two overlapping bands, 10–1610 Hz and 1–20 kHz, using identical preamplifier gain settings. Furthermore, because GR noise is strongly temperature dependent, additional measurements were conducted from 20 to 100 °C, enabling systematic separation of GR and CNF components across different thermal conditions.

## Results and discussion

### FD-SOI defects analysis method

**Fig. 1:**
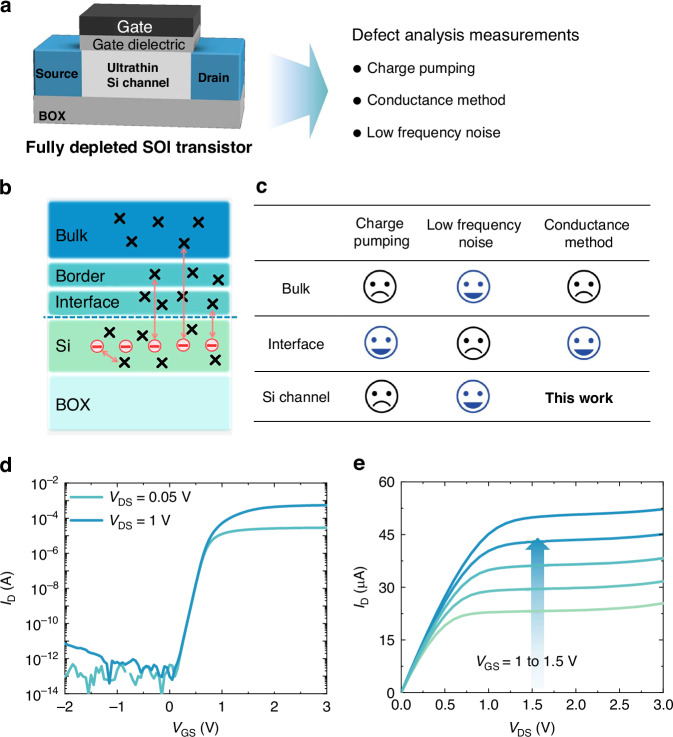
Overview of defect-sensitive characterization in an FD-SOI transistor. **a** Schematic of an FD-SOI transistor and the defect analysis techniques. **b** Illustration of trap distributions in the bulk oxide, interface, and silicon channel. **c** Comparison of the sensitivity of charge pumping, LFN, and conductance method measurements to traps in different regions. **d** Transfer characteristics (*I*_D_–*V*_GS_) of the FD-SOI transistor measured at different drain voltages. **e** Output characteristics (*I*_D_–*V*_DS_) showing the evolution of *I*_D_ with increasing *V*_G_

The FD-SOI transistors investigated in this work were fabricated using an industry-standard 80 nm CMOS process on 12-inch wafers by a commercial foundry service. As illustrated in Fig. 1a, the device consists of an ultra-thin silicon channel with a thickness of 9 nm formed on a BOX layer, together with a 5 nm SiO_2_ gate dielectric^27^. Owing to the fully depleted channel geometry, these FD-SOI transistors exhibit strong electrostatic control, suppressed short-channel effects, ultra-low off-state leakage current, and near-ideal SS^28,29^. In addition, carrier transport is enhanced by volume inversion in the ultra-thin silicon film, making this platform representative of advanced floating-body transistor technologies^30^.

Figure 1b schematically illustrates the spatial distribution of defects that may contribute to the electrical and noise characteristics of FD-SOI transistors. Possible defect locations include traps in the bulk oxide, interface traps at the Si/SiO_2_ interface, and defects inside the ultra-thin silicon channel. When inversion carriers are formed in the channel and transported from source to drain to establish the drain current, these carriers dynamically interact with defects located in the silicon channel, at the interface, and within the surrounding dielectric layers. Such defect–carrier interactions play a critical role in device reliability, as they can modulate carrier transport through trapping, detrapping, and GR processes.

To quantitatively analyze the intrinsic properties of these traps, including their density, energetic position, and characteristic time constants, various electrical characterization techniques have been developed, such as charge pumping, LFN spectroscopy, and the conductance method (Fig. 1c). Charge pumping measurements extract the interface trap density by periodically modulating the gate pulse and monitoring the recombination current flowing through the body terminal, which originates from carriers trapped and subsequently detrapped at the Si/SiO_2_ interface^31,32^. However, this approach cannot be directly applied to floating-body devices, as the absence of a body contact prevents access to the recombination current. LFN measurements analyze drain current fluctuations in the frequency domain, enabling identification of different noise mechanisms, such as GR noise, CNF noise, and mobility fluctuation noise^33–35^. Through LFN analysis, it is possible to profile trap distributions in the oxide bulk within a depth of approximately 1–2 nm from the interface and to extract the energetic position of deep-level defects in the silicon channel^36^. However, when extended to interface traps in gate dielectrics, the characteristic trap capture and emission time constants become too short to be reliably resolved within the bandwidth limitations of conventional noise measurement systems, rendering direct extraction impractical^37^. As a result, the conductance method has been introduced as an alternative technique for extracting interface trap density in floating-body devices. By analyzing the frequency-dependent conductance response, this method probes carrier capture and emission dynamics associated with interface traps through their contribution to the small-signal loss component of the device admittance, without requiring a body terminal^38^. However, the contribution of defects located inside the silicon channel to the small-signal admittance loss has not been systematically investigated (Fig. 1c).

Before discussing trap-related phenomena, the basic electrical characteristics of the FD-SOI transistor are first examined to confirm proper device operation. Figure 1d, e shows the transfer characteristics (*I*_D_–*V*_GS_) and output characteristics (*I*_D_–*V*_DS_), respectively. The transfer characteristics exhibit a monotonic increase in *I*_D_ with increasing gate voltage (*V*_G_) under nominal bias conditions, indicating well-controlled channel formation and stable transistor operation. The output characteristics demonstrate a clear modulation of the *I*_D_ with increasing *V*_DS_, followed by early current saturation at higher *V*_DS_, which is characteristic of fully depleted ultra-thin-body devices. In addition, the device shows a SS of approximately 70 mV/dec at room temperature, approaching the theoretical Boltzmann limit, confirming strong electrostatic control of the channel^39^. These results verify that the FD-SOI transistors operate reliably and provide a suitable platform for subsequent conductance and LFN analyses focused on defect-related carrier dynamics.

### Measurement of conductance method

**Fig. 2:**
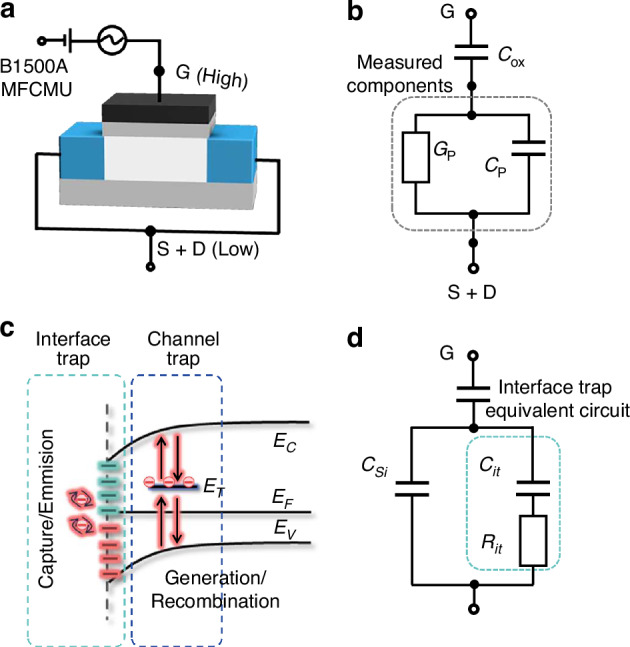
Measurement principle of the conductance method for trap analysis in an FD-SOI transistor. **a** Measurement configuration for the conductance method using a B1500A MFCMU. **b** Equivalent circuit representation of the measured admittance. The measured response consists of conductance *G*_p_ and a capacitance *C*_p_. **c** Schematic energy band diagram illustrating different defect-related carrier dynamics. Interface traps at the Si/SiO_2_ interface contribute to carrier capture and emission processes, whereas channel traps inside the silicon film induce generation and recombination dynamics. **d** Equivalent circuit model of interface traps. The interface trap response is modeled by a series *C*_it_-*R*_it_ network

As shown in Fig. 2a, conductance measurements were performed using a Keysight B1500A semiconductor parameter analyzer equipped with a multi-frequency capacitance measurement unit (MFCMU)^40^. During the measurement, the source and drain terminals were electrically shorted and connected to the low terminal, while the gate terminal was connected to the high terminal. The *C*–*f*/*G*–*f* measurements were performed from 1 kHz to 5 MHz using an AC small-signal amplitude of 20 mV RMS, while the DC gate bias was swept from 0.6 V to 1.0 V with a step of 0.1 V, as illustrated in Supplementary Fig. S1.

Figure 2b presents an equivalent circuit representation of the small-signal AC measurement. The measured components are expressed in terms of parallel conductance and capacitance components, denoted as *G*_p_ and *C*_p_, which are directly extracted from the AC response^41^. Before discussing the individual contributions of the measured conductance and capacitance components, Fig. 2c, d illustrates the physical dynamics and equivalent circuit model associated with interface traps. When inversion carriers are present in the channel, interface traps exchange carriers with the channel through capture and emission processes. These dynamics can be modeled by a capacitance associated with the trap occupancy, *C*_it_, in series with a resistance *R*_it_ that represents the finite capture and emission rates of the traps^42–44^. This series representation reflects the fact that the trap charge cannot respond instantaneously to the applied AC excitation, leading to a frequency-dependent impedance. Accordingly, the impedance of the interface trap can be expressed as a series combination of *C*_it_ and *R*_it_, shown below:

1 $${Z}_{{it}}={R}_{{it}}+\frac{1}{{jw}{C}_{{it}}}$$

This series impedance can be equivalently represented in terms of an interface-trap admittance, which may be decomposed into real and imaginary components. The real part of the admittance corresponds to the energy dissipation associated with carrier capture and emission, while the imaginary part reflects the delayed capacitive response of the trap occupancy. The resulting interface-trap admittance is therefore given by:

2 $${Y}_{{it}}=\frac{1}{{Z}_{{it}}}=\frac{{jw}{C}_{{it}}}{1+{jw}{\tau }_{{it}}}$$

Here, the characteristic time constant of the interface trap is ${\tau }_{{it}}={R}_{{it}}{C}_{{it}}$, which represents the finite response time associated with carrier capture and emission processes at the interface trap. The resulting interface-trap admittance is given by:

3 $${Y}_{{it}}=\frac{{w}^{2}{\tau }_{{it}}{C}_{{it}}}{1+{\left(w{\tau }_{{it}}\right)}^{2}}+j\frac{w{C}_{{it}}}{1+{\left(w{\tau }_{{it}}\right)}^{2}}$$

Accordingly, the real and imaginary components of the interface-trap admittance can be expressed as:

4 $${{Re}(Y}_{{it}})={G}_{{it}}=\frac{{w}^{2}{\tau }_{{it}}{C}_{{it}}}{1+{\left(w{\tau }_{{it}}\right)}^{2}}$$

5 $${{{Im}(Y}_{{it}})=C}_{{it}}=j\frac{w{C}_{{it}}}{1+{\left(w{\tau }_{{it}}\right)}^{2}}$$

Here, the real part of the interface-trap admittance corresponds directly to the conductance component measured in the AC experiment, denoted as *G*_p_ in Fig. 2b. Under this framework, the interface-trap capacitance *C*_it_ is related to the interface trap density through the relation ${C}_{{it}}={{qD}}_{{it}}$, where q is the elementary charge and *D*_it_ represents the interface trap density per unit energy. Based on these considerations, the frequency-dependent interface trap density *D*_it_ with its ${\tau }_{{it}}$, can be extracted using the following model^18^:

6 $$\frac{{G}_{p}}{w}={{qD}}_{{it}}\frac{w{\tau }_{{it}}}{1+{\left(w{\tau }_{{it}}\right)}^{2}}$$

However, as illustrated in Fig. 2c, defects located inside the silicon channel can also participate in carrier GR processes, giving rise to additional loss mechanisms that may appear in the form of a conductance component in AC measurements. In fully depleted ultra-thin channels, these channel-related GR processes can modulate the carrier population dynamically and introduce frequency-dependent dissipation similar to that attributed to interface traps. Despite this possibility, the conventional conductance framework implicitly assumes that the measured conductance originates solely from interface-trap-related capture and emission dynamics. The contribution of silicon channel traps to the small-signal conductance response has not been investigated. As a result, when channel defects are present, the conductance extracted using Eq. (6) may reflect a superposition of interface and channel defect dynamics, rather than a pure interface-trap response.

### Conductance method measurements of bulk and FD-SOI MOSFETs

**Fig. 3:**
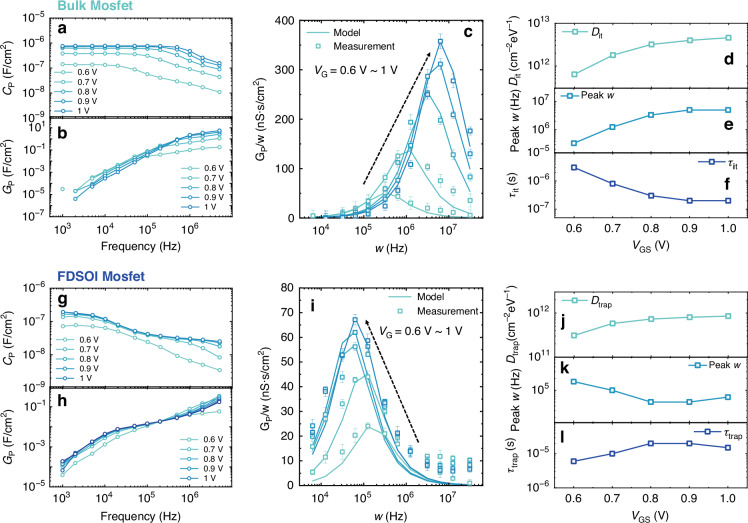
Frequency-dependent conductance and capacitance characteristics of bulk and FD-SOI MOSFETs measured using the conductance method. **a**, **b** Measured capacitance *C*_p_ and conductance *G*_p_ as a function of frequency for the bulk MOSFET at different *V*_G_s. **c** *G*_p_/*ω* spectra of the bulk MOSFET, measured from five devices sampled at representative locations across the 12-inch wafer, where the solid lines represent the measured data and the symbols denote the corresponding model fitting curves, showing a *V*_G_ dependent shift in both the peak frequency and peak magnitude. **d**–**f** *V*_G_ dependence of the extracted interface trap density *D*_it_, peak angular frequency, and trap time constant for the bulk MOSFET. **g**, **h** Measured *C*_p_ and *G*_p_ as a function of frequency for the FD-SOI MOSFET. **i** *G*_p_/*ω* spectra of the FD-SOI MOSFET, collected from five devices distributed across representative regions of the 12-inch wafer, exhibiting a weak dependence of the peak frequency on *V*_G_. **j**–**l** Gate-voltage dependence of the extracted trap density, peak angular frequency, and effective trap time constant for the FD-SOI MOSFET

Figure 3a–f presents the conductance analysis results obtained from a conventional bulk MOSFET, while Fig. 3g–l shows the corresponding results for the FD-SOI device. For the conductance measurements, both the FD-SOI and bulk MOSFET devices had a channel width and length of *W*/*L* = 100/100 μm. We first focus on the bulk MOSFET results. Figure 3a and b show the frequency dependence of the measured capacitance *C*_p_ and conductance *G*_p_ for *V*_G_s ranging from 0.6 V to 1.0 V. Figure 3c shows the normalized conductance *G*_p_ divided by the angular frequency (*ω* = 2π*f*) as a function of frequency for different *V*_G_s obtained from five devices selected from spatially distributed locations across the 12-inch wafer, as illustrated in Supplementary Fig. S2. In Fig. 3c, the solid lines represent the experimentally measured *G*_p_/*ω*, while the discrete symbols correspond to the fitting results obtained using the interface-trap conductance model described by Eq. (6). The *G*_p_/*ω* exhibits characteristic bell-shaped behavior, which is a hallmark of interface-trap-dominated conductance in bulk MOSFETs. As the *V*_G_ increases from 0.6 V to 1.0 V, both the peak frequency and the peak magnitude of the *G*_p_/*ω* curves systematically increase. This behavior reflects the distribution and dynamic response of interface traps under varying surface potential conditions. With increasing *V*_G_, the surface potential increases and the inversion carrier density near the Si/SiO_2_ interface becomes higher, leading to an enhanced probability of carrier capture and emission at interface traps. As a result, the effective trap time constant decreases, which manifests as a shift of the peak frequency toward higher values. In addition, the peak magnitude of the *G*_p_/*ω* increases with *V*_G_, indicating a gate-voltage-dependent activation of interface trap states. As the surface potential changes, band bending at the interface modifies the energetic alignment between the Fermi level and the interface trap distribution, thereby altering the number of active trap states that can participate in carrier exchange. Conversely, if the peak magnitude were independent of *V*_G_, it would imply that the effective interface trap density contributing to the conductance response remains constant, providing evidence that the interface trap population is insensitive to surface potential modulation. Figure 3d–f summarize the quantitative parameters extracted from the *G*_p_/*ω* analysis as a function of *V*_G_. Figure 3d shows the extracted interface trap density *D*_it_. Figure 3e and f present the corresponding peak *ω* and the effective trap time constant, respectively.

We now turn to the conductance analysis results obtained from the FD-SOI device. Figure 3g and h show the frequency dependence of the measured capacitance *C*_p_ and conductance *G*_p_ for different *V*_G_s. Frequency response of *C*_p_ and *G*_p_ differ from those observed in the bulk MOSFET. Figure 3i presents the normalized conductance *G*_p_ divided by *ω* as a function of frequency for *V*_G_s ranging from 0.6 V to 1.0 V measured from five devices sampled at representative locations across the 12-inch wafer, as shown in Supplementary Fig. S2. Similar to the bulk device, the *G*_p_/*ω* exhibit a bell-shaped behavior and can be reasonably fitted using the conductance model described by Eq. (6). However, a key difference is observed in the gate-voltage dependence of the extracted features. As the *V*_G_ increases, the peak magnitude of the *G*_p_/*ω* curves increases, whereas the peak frequency remains nearly unchanged over the investigated *V*_G_ range. Figure 3j–l summarize the quantitative parameters extracted from the *G*_p_/*ω* analysis as a function of *V*_G_. Figure 3j shows the extracted trap density *D*_trap_, while Fig. 3k and l present the corresponding peak *ω* and effective trap time constant, respectively. Notably, the weak *V*_G_ dependence of peak frequency and trap time constant contrasts with the behavior observed in the bulk MOSFET, indicating that the conductance response in the FD-SOI device follows a trend distinct from that expected for conventional interface-trap-dominated capture and emission dynamics.

Meanwhile, to evaluate the influence of parasitic components associated with the body terminal, additional conductance measurements were performed under floating-ground conditions for both the bulk MOSFET and the FD-SOI device, as shown in Supplementary Fig. S3. The frequency dependences of *C*_p_, *G*_p_, and the normalized conductance *G*_p_/*ω* were analyzed and compared with those obtained under ground-body conditions (Supplementary Fig. S3 and Fig. 3). In both device types, the overall spectral shapes and gate-voltage trends remain essentially unchanged when the body terminal is floated. This consistent behavior indicates that the observed conductance response is not significantly affected by parasitic components related to the body terminal, and therefore, the body-induced parasitic effects can be considered negligible in the present measurements.

Although the body-induced parasitic effects were found to be negligible under the present measurement conditions, the conductance method can also be influenced by overlap capacitance and stray parasitic components^16^. In this work, no explicit correction or de-embedding procedure was performed for these effects. Therefore, the trap-related parameters extracted from the conductance spectra should be regarded as effective values obtained from the measured admittance response. The main purpose of the present analysis is not to provide a fully de-embedded extraction of absolute interface trap density, but to clarify the dominant physical origin of the conductance response associated with interface- and channel-defect-related carrier dynamics in FD-SOI transistors.

### Low-frequency noise characteristics of FD-SOI devices

To elucidate the origin of the anomalous *G*_p_/*ω* characteristics observed in the FD-SOI device, which deviate from the conventional interface-trap-dominated behavior of bulk MOSFETs, LFN measurements were systematically performed. Before discussing the LFN characteristics, the temperature-dependent transfer and output characteristics of the FD-SOI device are shown in Supplementary Fig. S4. As shown in Supplementary Fig. S4a, the transfer characteristics exhibit an increase in the subthreshold drain current and a slight degradation of the SS with increasing temperature. In addition, the output characteristics in Supplementary Fig. S4b show a slight increase in drain current at elevated temperatures. This increase can be attributed to thermally activated carrier generation and the temperature-induced reduction of the effective threshold voltage, which outweigh the mobility degradation caused by enhanced phonon scattering under the present measurement conditions. These temperature-dependent electrical characteristics provide the fundamental device behavior corresponding to the conductance and LFN analyses discussed below.

**Fig. 4:**
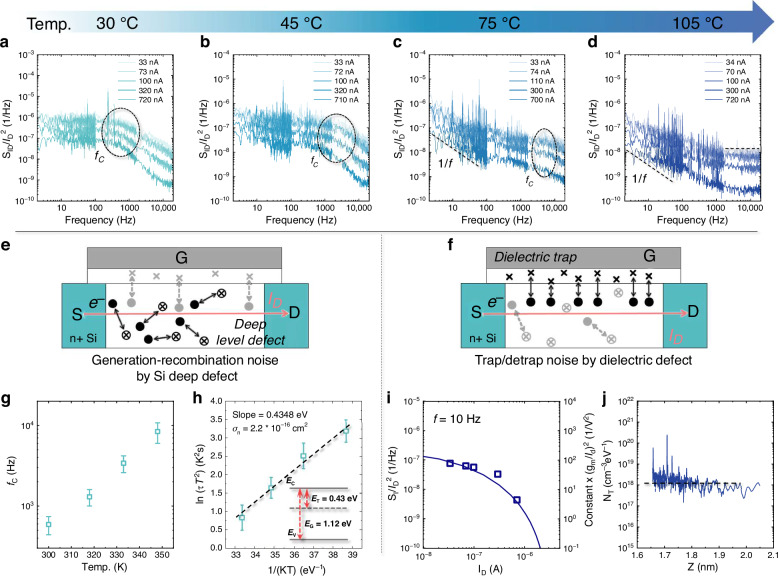
Temperature-dependent low-frequency noise characteristics of the FD-SOI MOSFET. **a**–**d** *I*_D_ noise PSD as a function of frequency measured at different temperatures. **e**, **f** Schematic illustrations of the dominant noise mechanisms: GR noise associated with deep-level defects in the silicon channel and trapping/detrapping noise induced by dielectric defects. **g** Extracted *f*_c_ as a function of temperature. **h** Arrhenius analysis of the temperature dependence of the *f*_c_, indicating a thermally activated trap process. **i** *I*_D_ dependence of the noise power spectral density with fitting using the (*g*_m_/*I*_D_)^2^. **j** Depth-dependent distribution of the extracted trap density

Figure 4a–d shows the *I*_D_-normalized PSD measured at different temperatures under various *I*_D_ conditions. The LFN characteristics shown in Fig. 4 were measured under the body-ground condition. At room temperature, a Lorentzian-type noise component is observed as the drain current increases. In general, for Lorentzian noise originating from random telegraph noise (RTN) associated with interface trap capture and emission, an increase in drain current or gate voltage enhances the trap and detrap probability, leading to a reduction in the effective time constant and a corresponding shift of the corner frequency (*f*_c_) toward higher values in the PSD^45,46^. Such behavior reflects the strong dependence of interface-trap dynamics on surface potential and inversion carrier density.

However, as shown in Fig. 4a, the extracted *f*_c_ does not vary with *I*_D_ or *V*_G_. Instead, the *f*_c_ remains nearly constant over the current range investigated and becomes clearly observable only as the temperature increases. This behavior is inconsistent with interface-trap-induced RTN and is instead characteristic of GR noise originating from deep-level defects inside the silicon channel (Fig. 4e). In this case, the characteristic time constant is governed primarily by the intrinsic properties of the channel traps, rather than by carrier density modulation. The GR noise physics model is given by the following expression^47^:

7 $$\frac{{S}_{{I}_{D}}}{{{I}_{D}}^{2}}={\frac{{{q}^{2}F}_{t}(1-{F}_{t}){N}_{T,{GR}}}{{LW}{C}_{{OX}}}\left(\frac{{g}_{m}}{{I}_{D}}\right)}^{2}\times \frac{4\tau }{1+4{\pi }^{2}{\tau }^{2}{f}^{2}}$$

where *g*_m_ is the transconductance, *F*_t_ is fractional occupancy of traps around the Fermi level and *N*_T,GR_ is the volume trap density in the film, *C*_OX_ is the oxide capacitance per unit area, *q* is the electron charge, *W* and *L* represent the width and the length of the FET channel and *τ* is carrier time constant. Using this model, the extracted volume trap density associated with the GR component, *N*_T,GR_, is approximately 7.35 × 10^17^ cm^–3^eV^–1^. To quantitatively analyze the intrinsic properties of the silicon channel traps responsible for the observed GR noise, Fig. 4g shows the temperature dependence of the extracted corner frequency. Based on this temperature-dependent behavior, an Arrhenius plot can be constructed using the relation^48^:

8 $$\tau =\left(\frac{1}{2\pi {f}_{c}}\right)=\left(\frac{1}{{c}_{n}\left(n\left(x,y\right)+{n}_{t}\right)+{c}_{p}\left(p\left(x,y\right)+{p}_{t}\right)}\right)$$

9 $${ln}\left(\tau {T}^{2}\right)=\left(\frac{{E}_{c}-{E}_{t}}{{KT}}\right)+\left(\frac{1}{{c}_{n}\left(n\left(x,y\right)+{n}_{t}\right)+{c}_{p}\left(p\left(x,y\right)+{p}_{t}\right)}\right)$$

where *c*_*n*_ and *c*_*p*_ represent the capture rates for electrons and holes, respectively, which are defined as the product of the thermal velocity and the capture cross section for electrons (*σ*_*n*_) or holes (*σ*_*p*_). The terms *n*(*x,y*) and *p*(*x,y*) denote the local free carrier concentrations at the trap position (*x,y*) within the depletion region of the transistor. In this context, *h* is Planck’s constant; *m*_*e*_ and *m*_*h*_ are the effective masses of electrons and holes, respectively; and *M*_*C*_ refers to the number of conduction band minimum. By analyzing the slope and intercept of a plot of *ln(τT*^2^*)* versus 1/*kT*, one can determine the energy difference between the band edge and the trap level (i.e., Δ*E* = *E*_C_ − *E*_T_) and extract the electron capture cross section (*σ*_*n*_) of the trap. From the Arrhenius plot shown in Fig. 4h, the slope corresponds to an energy separation of *E*_C_ − *E*_T_ = 0.4348 eV, indicating the energetic position of the dominant deep-level trap inside the silicon channel. In addition, the y-intercept yields an electron capture cross section of approximately 2.2 × 10^–16 ^cm^2^ for the extracted trap.

As the temperature increases, a clear transition in the noise spectrum is observed, as shown in Fig. 4c, d. The low-frequency region evolves from a Lorentzian plateau to a 1/*f*-type noise behavior. To analyze the origin of the 1/*f* noise component, the PSD extracted at 10 Hz was plotted as a function of (*g*_m_/*I*_D_)^2^, as shown in Fig. 4i. These characteristics indicate the observed 1/*f* noise follows the CNF model^49,50^.

10 $$\frac{{S}_{{I}_{D}}}{{{I}_{{\rm{D}}}}^{2}}={\left(\frac{{g}_{{\rm{m}}}}{{I}_{{\rm{D}}}}\right)}^{2}{S}_{{\rm{Vfb}}}$$

where

11 $${S}_{{V}_{{fb}}}=\frac{{q}^{2}{kT}{\lambda N}_{T}}{{fWL}{{C}_{{OX}}}^{2}}$$

where *g*_*m*_ is the transconductance, *S*_Vfb_ is the flat band voltage fluctuation, *C*_OX_ is the oxide capacitance per unit area, *q* is the electron charge, *T* is the temperature in Kelvin, λ is the oxide tunneling distance, *N*_T_ is the volume trap density, *W* and *L* represent the width and the length of the FET channel. According to this model, the noise originates from carrier trapping and detrapping processes occurring in defects distributed within the gate dielectric (Fig. 4f). Unlike channel deep-level traps, the intrinsic properties of dielectric traps are not strongly modulated by temperature over the investigated range. Therefore, the CNF analysis enables quantitative profiling of the *N*_T_ inside the dielectric. The spatial position z-axis of the dielectric traps as a function of frequency can be expressed using the following relation^51^.

12 $$z=\lambda \mathrm{ln}\left(\frac{1}{2\pi f\tau }\right)$$

where τ is the time for tunneling into a trap state (10^−10^s) at the interface (z = 0). Figure 4j shows the extracted trap volume density as a function of depth in the dielectric. The extracted trap density is on the order of 2 × 10^18 ^cm^–3^ eV^–1^.

Meanwhile, to examine whether the floating-body configuration induces additional noise amplification through the body-related RC network, output characteristics and LFN spectra were additionally measured under floating body conditions, as shown in Supplementary Fig. S5^52^. The output characteristics measured under body-ground and floating body conditions do not exhibit a distinct kink effect, indicating that significant floating body charging is not induced under the present bias conditions. Moreover, the normalized drain-current noise PSDs obtained under floating body conditions show no noticeable enhancement, additional low-frequency component, or RC-network-related spectral feature compared with those obtained under body-ground conditions. The overall PSD magnitude, Lorentzian-type GR component, and temperature-dependent evolution of the spectra remain nearly unchanged between the two body-bias conditions. These results indicate that floating-body-induced shot-noise amplification associated with the body-related RC network is negligible in the present measurements. Therefore, the LFN characteristics analyzed in this work are mainly attributed to defect-mediated GR dynamics in the ultra-thin silicon channel rather than to floating-body RC-network-induced noise amplification.

### Conductance method measurement with temperature

**Fig. 5:**
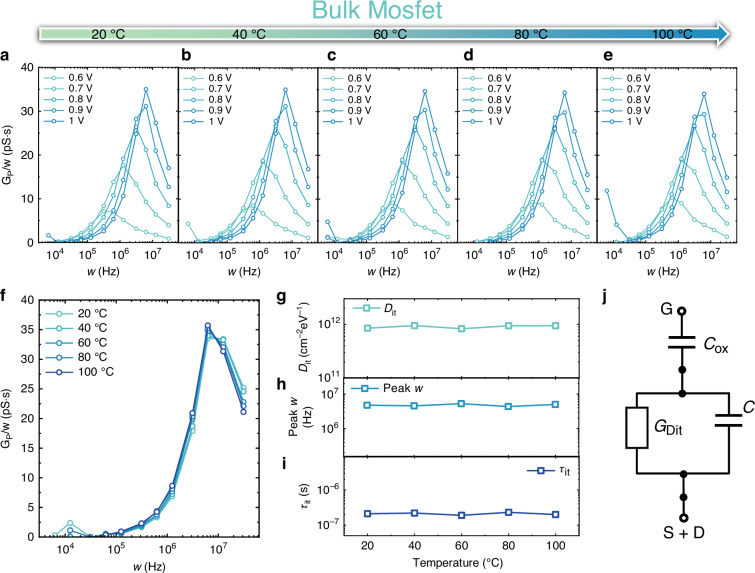
Temperature-dependent conductance analysis of the bulk MOSFET using the conductance method. **a**–**e** *G*_p_/*ω* measured at different *V*_G_s for temperatures ranging from 20 to 100 °C. **f** Comparison of *G*_p_/*ω* at different temperatures, showing similar peak positions and magnitudes. **g**–**i** Temperature dependence of the extracted interface trap density *D*_it_, peak angular frequency, and effective trap time constant. **j** Equivalent circuit used for the conductance analysis, consisting of the capacitance *C* and the interface trap conductance *G*_Dit_

To examine the temperature dependence of the conductance response originating from interface traps, the *G*_p_/*ω* characteristics of a conventional bulk MOSFET were first analyzed as a function of temperature, as shown in Fig. 5a–e. Since the intrinsic properties of interface traps, such as their energy distribution, density and capture cross section, are not strongly modulated over this temperature range, the characteristic bell-shaped *G*_p_/*ω* behavior remains nearly unchanged from 20 °C to 100 °C, as summarized in Fig. 5f. Supplementary Fig. S6 shows the temperature-dependent *C*_p_ and *G*_p_ characteristics of the bulk MOSFET measured over the frequency range from 20 to 100 °C. Despite the change in temperature, the overall frequency response and gate-voltage trends of both *C*_p_ and *G*_p_ remain unchanged. Figure 5g–i presents the extracted interface trap density *D*_it_, peak *ω*, and effective trap time constant as a function of temperature, respectively, all of which exhibit minimal temperature dependence. This behavior is consistent with the conventional interface-trap-dominated conductance response. The corresponding equivalent circuit, shown in Fig. 5j, consists of the *C*_ox_ in parallel with the interface-trap conductance component, where the measured conductance originates solely from interface-trap-related capture and emission dynamics.

**Fig. 6:**
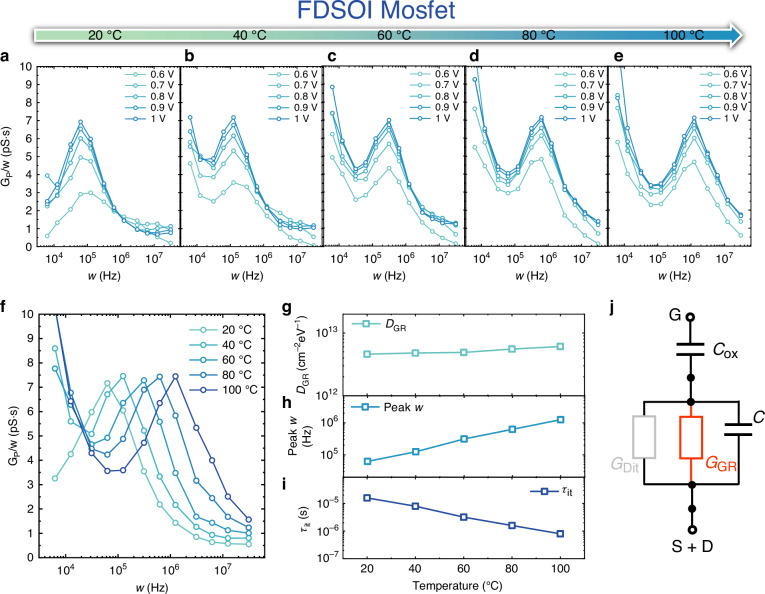
Temperature-dependent conductance analysis of the FD-SOI MOSFET using the conductance method. **a**–**e** *G*_p_/*ω* measured at different *V*_G_s for temperatures ranging from 20 to 100 °C. **f** Comparison of *G*_p_/*ω* at different temperatures, showing a pronounced temperature dependence of both peak position and magnitude. **g**–**i** Temperature dependence of the extracted trap-related parameters, including the effective trap density *D*_GR_, peak angular frequency, and effective trap time constant. **j** Equivalent circuit used for the conductance analysis, where the total conductance includes contributions from both interface traps (*G*_Dit_) and GR processes in the silicon channel (*G*_GR_)

In contrast, Fig. 6a–e presents the temperature-dependent *G*_p_/*ω* characteristics of the FD-SOI device as a function of frequency. For all *V*_G_ conditions, the peak position systematically shifts toward higher frequencies as the temperature increases. This behavior is consistent with the temperature-dependent corner-frequency shift observed in the previous LFN analysis, indicating a common underlying physical origin. Figure 6f summarizes the *G*_p_/*ω* at different temperatures, clearly showing the temperature-induced shift of the peak position. Supplementary Fig. S7 presents the temperature-dependent *C*_p_ and *G*_p_ characteristics of the FD-SOI measured from 20 °C to 100 °C, showing noticeable variations in the frequency response as the temperature changes. Figure 6g–i presents the extracted GR trap density *D*_GR_, peak ω, and effective trap time constant as a function of temperature, respectively. The pronounced temperature dependence of these parameters suggests that the dominant contribution to the *G*_p_/*ω* response does not originate from interface traps, but rather from deep-level defects within the silicon channel. As illustrated in the equivalent circuit in Fig. 6j, the conductance component is therefore dominated by GR processes associated with silicon deep-level defects, rather than by interface-trap-related capture and emission dynamics.

Although interface traps may also contribute to the measured conductance response in the FD-SOI device, a distinct double-peak feature is not observed under the present measurement conditions. This can be understood from the relative magnitude of the interface-trap-related and GR-related contributions. As a first-order approximation, the effective two-dimensional interface-trap density contributing to the conductance response can be estimated from the near-interface trap density extracted by CNF-based LFN analysis as^53^:

13 $${D}_{{it}}=\lambda {N}_{T}$$

where ${N}_{T}$ is the volume trap density extracted from the CNF-based LFN analysis and λ is the effective tunneling attenuation length, which converts the near-interface oxide trap density into an equivalent two-dimensional trap density participating in the conductance response. Using this first-order approximation, the effective two-dimensional interface-trap density is estimated to be approximately 2 × 10^10 ^cm^–2^eV^–1^. In contrast, the GR trap density extracted from the conductance analysis in Fig. 3j is on the order of 5 × 10^12 ^cm^–2^eV^–1^. This difference of more than one order of magnitude indicates that the GR admittance loss is substantially larger than the interface-trap-related conductance response. Therefore, although the interface-trap response may exist in the FD-SOI device, it is likely masked by the much stronger GR contribution and is not resolved as an independent peak. If the silicon channel did not contain dominant GR defects, the conductance spectra would be expected to mainly reflect the interface-trap response. However, in the present ultra-thin FD-SOI device, the large GR contribution results in a single dominant bell-shaped conductance peak rather than a clearly resolved double-peak structure.

This dominance of the GR response is further supported by the volume-inversion nature of the ultra-thin FD-SOI channel due to strong quantum confinement effects. In this condition, inversion carriers are not confined only at the Si/SiO_2_ interface but are distributed throughout the silicon body, with the carrier centroid located inside the channel. As a result, carrier transport becomes more strongly influenced by defects present within the silicon channel rather than by interface traps alone. Consequently, current fluctuations originating from silicon channel defects can become more dominant than those caused by interface trap capture and emission processes. Moreover, since the number of inversion carriers in an ultra-thin channel is finite, the conductance response becomes dependent on the traps that have the highest interaction probability with the carriers. Under such conditions, the overall measured conductance response is predominantly determined by the stronger interaction between inversion carriers and defects located inside the silicon channel.

Additionally, the GR trap density extracted using the conductance method can be expressed as follows:

14 $$\frac{{G}_{P,{GR}}}{w}={{qD}}_{{GR}}\frac{w{\tau }_{{GR}}}{1+{\left(w{\tau }_{{GR}}\right)}^{2}}$$

Here, *D*_GR_ is expressed in a two-dimensional form, and it can be converted into a volume trap density by incorporating the thickness of the silicon channel.

15 $${D}_{{GR}}={N}_{T,{GR}}{T}_{{Si}}$$

Based on these equations, the extracted volume density of the GR traps, *N*_T,GR_, is 8.89 × 10^17 ^cm^–3^eV^–1^. This value is in good agreement with the trap density independently extracted from the GR noise model in the previous LFN analysis, indicating that both the conductance and noise measurements consistently point to the presence of deep-level defects inside the silicon channel. Such consistency further supports the conclusion that the dominant conductance response in the FD-SOI device originates from silicon channel traps rather than conventional interface-trap dynamics.

Based on this consistency between conductance and LFN analyses, the channel-defect-dominated conductance behavior observed in this work is not necessarily restricted to the specific 9 nm silicon body thickness^54^. Rather, similar behavior can be expected in a broader ultra-thin-body regime when GR defects are present inside the silicon channel. Even for a relatively thicker silicon body, the GR-related response can still become dominant if the effective contribution of GR-active channel defects is larger than that of interface traps. Therefore, the dominant conductance response is not determined by the silicon body thickness alone, but by the relative contribution of interface traps and channel defects. When the interface-trap contribution is dominant, the conductance spectra are expected to follow the conventional interface-trap capture/emission behavior. In contrast, when LFN analysis reveals a clear GR noise component associated with channel defects and the effective channel-defect contribution exceeds the interface-trap contribution, the GR-related admittance loss can dominate the measured conductance response. In the present FD-SOI device, the clear GR component identified by LFN analysis and the larger effective channel-defect contribution indicate that the measured conductance response is predominantly governed by channel-defect-related GR dynamics rather than by conventional interface-trap capture/emission.

Meanwhile, possible electrostatic coupling through the BOX or the back interface should also be considered in FD-SOI devices^17^. Previous studies have reported that, when the BOX is sufficiently thin and channel defects are not dominant, back-gate bias modulation can be used to separately probe the front- and back-interface trap responses. In the present FD-SOI device, however, the BOX thickness is 200 nm, which is relatively thick for strong back-gate coupling. Thus, the electrostatic influence of the substrate or back interface on the front-gate conductance response is expected to be weak. Therefore, the observed conductance response is mainly discussed in terms of front-gate-controlled carrier dynamics and channel-defect-related GR processes.

## Conductance method measurement of stressed FD-SOI

**Fig. 7:**
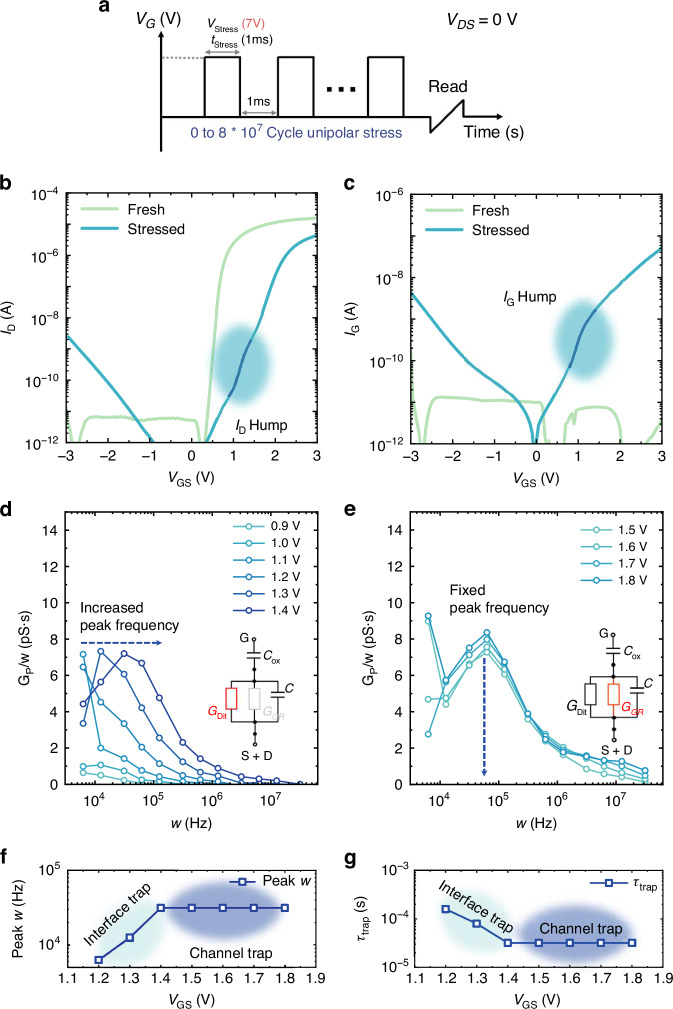
Electrical degradation and conductance response of the FD-SOI MOSFET under unipolar gate stress. **a** Gate stress waveform used for reliability testing, consisting of repeated unipolar voltage pulses followed by a read operation at zero drain bias. **b** Transfer characteristics showing the emergence of *I*_D_ hump after stress. **c** *I*_G_ characteristics exhibiting a pronounced hump in the stressed device. **d** *G*_p_/*ω* measured at different *V*_G_s below the hump regime, where the peak frequency shifts with *V*_G_. **e** *G*_p_/*ω* measured at higher *V*_G_s above the hump regime, showing fixed peak frequency. Extracted (**f**) peak angular frequency and (**g**) trap time constant as a function of gate bias after electrical stress

Figure 7a shows the cycling stress conditions applied to induce degradation in the FD-SOI device. A unipolar gate pulse of 7 V was applied for up to 8 × 10⁷ cycles. Figure 7b and c present the transfer characteristics of the fresh and stressed devices, showing the *I*_D_ and gate current (*I*_G_), respectively. After stress, a distinct hump appears in both the drain and gate current characteristics. The corresponding conductance responses of the degraded device are shown in Fig. 7d, e. Figure 7d presents the *G*_p_/*ω* in the *V*_G_ range where the hump appears (0.9–1.4 V). In this region, the peak frequency shifts with increasing *V*_G_, which is consistent with the typical response of interface-trap-dominated capture and emission dynamics^55^. In contrast, Fig. 7e shows the *G*_p_/*ω* characteristics at *V*_G_s beyond the hump region. In this regime, the peak frequency remains nearly constant even as the *V*_G_ increases. This behavior deviates from the interface-trap response and instead indicates that the conductance is dominated by GR processes associated with deep-level defects in the silicon channel. Figure 7f and g summarize the extracted peak frequency and trap time constant, respectively, further confirming the bias-dependent transition from interface-trap-dominated behavior in the hump region to channel-defect-dominated GR dynamics at higher gate biases.

This behavior can be understood from the formation of volume inversion in the ultra-thin silicon channel. At relatively low *V*_G_, the inversion carriers are not yet fully centered within the silicon body. Under this condition, the additional interface traps generated during the degradation process have a higher probability of interacting with the carriers. These newly generated interface traps introduce an additional *C*_it_, which leads to the hump observed in the transfer characteristics and produces a *V*_G_ dependent shift of the peak frequency in the conductance response. However, once the *V*_G_ exceeds the hump region, a well-developed volume inversion is formed in the ultra-thin silicon channel, and the carrier centroid moves further into the silicon body. In this regime, the finite number of inversion carriers is more likely to interact with the pre-existing deep-level defects inside the silicon channel than with the interface traps. As a result, the conductance response becomes dominated by the channel-related GR processes, and the peak frequency remains nearly unchanged with increasing *V*_G_.

Although the stressed conductance response indicates the generation of additional interface traps, the possibility that electrical stress also affects pre-existing channel defects should also be considered. In the higher-gate-bias regime, where the channel-defect-related GR response is dominant, the peak frequency remains nearly fixed and is close to that of the fresh FD-SOI device (Fig. 3i). This suggests that the intrinsic time constant of the dominant channel GR process (trap) is not significantly altered by the applied stress. To further examine the stress-induced defect response, stress-cycle-dependent LFN measurements were performed using an FD-SOI device with *W*/*L* = 1/0.52 μm, as shown in Supplementary Fig. S8. As the stress cycles increase, the transfer characteristics exhibit degraded SS, indicating stress-induced defect generation. In the corresponding LFN spectra, the fresh device shows a Lorentzian-type GR noise component, whereas the stressed devices show a gradual formation of a sloped low-frequency component before the corner frequency, together with a reduced PSD magnitude near the corner frequency. This behavior indicates that, as oxide/interface trap states increase during electrical degradation, the finite inversion carriers interact not only with pre-existing silicon channel traps but also with the newly generated oxide/interface trap states. However, the corner-frequency position remains nearly unchanged with increasing stress cycles. Since the corner frequency is associated with the characteristic time constant of the GR-active channel defect, this result suggests that the stress-induced degradation mainly increases oxide/interface trap contributions, while the intrinsic dynamics of the silicon channel GR defect are not significantly modified. A more detailed discussion of the stress-induced evolution of the LFN spectra and its relation to oxide/interface trap generation is provided in previous work^21^. Therefore, stress-induced degradation is mainly associated with oxide/interface trap generation, while the pre-existing channel defect continues to determine the GR-related characteristic frequency.

## Conclusions

We demonstrated that the conductance response in fully depleted SOI transistors is not governed solely by interface traps but is strongly influenced by GR processes associated with deep-level defects inside the ultra-thin silicon channel. By combining frequency-dependent conductance measurements with temperature-dependent LFN spectroscopy, we systematically clarified the physical origin of the *G*_p_/*ω* characteristics observed in floating-body transistors. While bulk MOSFETs exhibit the expected interface-trap-dominated conductance behavior, FD-SOI transistors show gate-voltage-independent yet strongly temperature-dependent conductance peaks. The analysis confirms that this behavior originates from thermally activated GR dynamics in the silicon channel, and the trap density extracted from the conductance method is in good agreement with values independently obtained from the noise model. Furthermore, electrical stress induces a bias-dependent transition in the dominant conductance mechanism, revealing a crossover from interface-trap-dominated to channel-defect-dominated dynamics. These results indicate that conventional interface-trap-only interpretations of the conductance method can lead to misestimation of defect properties in ultra-thin floating-body devices. The present study provides a physically consistent framework for interpreting conductance measurements and offers practical guidelines for reliable defect characterization in advanced logic and memory technologies.

## Supplementary information

Supplementary Information

## Data Availability

The datasets used and/or analyzed during the current study are available from the corresponding author on reasonable request.
