# Supplementary material for: Defect-mediated generation–recombination dynamics governing conductance response in floating-body transistors
Source: Microsyst Nanoeng. 2026 Aug 4;12:285. doi: 10.1038/s41378-026-01393-z (PMC13434757; doi:10.1038/s41378-026-01393-z)
Supplement: Supplementary file 1 [file 41378_2026_1393_MOESM1_ESM.docx]

**Supporting Information**

Defect-Mediated Generation–Recombination Dynamics Governing Conductance Response in Floating-Body Transistors

Been Kwak^1†^, Changhyeon Han^1†^, Hwoibin You^1†^_,_ Wongi Hong^1†^, Joonhyeok Choi^1^, Youngchan Cho^2^, Sangwan Kim^3^, Wonjun Shin^2,3*^, Daewoong Kwon^1*^

^†^B. Kwak, C. Han, H. You and W. Hong contributed equally to this work

^1^Department of Electrical Engineering, Hanyang University, Seoul 04763, Korea

^2^Department of Semiconductor Convergence Engineering, Sungkyunkwan University, Suwon 16419, Republic of Korea

^3^Department of Electrical and Computer Engineering, Sungkyunkwan University, Suwon 16419, Republic of Korea

*Correspondence: swj0107@skku.edu (W. Shin), and dw79kwon@hanyang.ac.kr (D. Kwon).

The following files are available free of charge.
Figures S1 ~ S8

**Figure S1.** Schematic waveform used for the *C*–*f*/*G*–*f* conductance measurements. The conductance measurements were performed over a frequency range from 1 kHz to 5 MHz using an AC small-signal amplitude of 20 mV RMS. The DC gate bias was swept from 0.6 V to 1.0 V with a step of 0.1 V.

**Figure S2.** Schematic of the device locations selected for device-to-device variation analysis on a 12-inch FD-SOI wafer.

**Figure S3.** Frequency-dependent capacitance (*C*_p_), conductance (*G*_p_), and normalized conductance (*G*_p_/ω) characteristics of (a)-(c) bulk MOSFET and of (d)-(f) FD-SOI devices under ground-body conditions.

**Figure S4.** Temperature-dependent transfer and output characteristics of the FD-SOI device. (a) *I*_D_-*V*_GS_ characteristics measured at different temperatures. (b) *I*_D_-*V*_DS_ characteristics measured at different temperatures.

**Figure S5.** (a) Output characteristics of the FD-SOI device measured under body-ground conditions at *V*_GS_ = 1 V for different temperatures. (b–d) Normalized *S*_ID_/*I*_D_^2^ measured under body-ground conditions at 20°C, 50°C, and 100°C, respectively. (e) Output characteristics measured under body-floating conditions at *V*_GS_ = 1 V for different temperatures. (f–h) Corresponding *S*_ID_/*I*_D_^2^ measured under body-floating conditions at 20°C, 50°C, and 100°C, respectively.

**Figure S6.** Temperature-dependent capacitance (*C*_p_) and conductance (*G*_p_) characteristics of the bulk MOSFET. (a–e) Frequency-dependent *C*_p_ at temperatures from 20°C to 100°C. (f–j) Corresponding *G*_p_ characteristics as a function of frequency.

**Figure S7.** Temperature-dependent capacitance (*C*_p_) and conductance (*G*_p_) characteristics of the FD-SOI MOSFET. (a–e) Frequency-dependent *C*_p_ at temperatures from 20°C to 100°C. (f–j) Corresponding *G*_p_ characteristics as a function of frequency.

**Figure S8.** (a) Gate-stress waveform used for stress-cycle. (b) Transfer characteristics of the FD-SOI device after different stress cycles. (c-e) Normalized drain-current noise PSDs measured after different stress cycles.
